# Gender relations and women’s empowerment in small-scale irrigated forage production in Ethiopia

**DOI:** 10.1371/journal.pone.0309927

**Published:** 2024-12-23

**Authors:** Immaculate Omondi, Esther Njuguna-Mungai, Melkamu Bezabih Derseh, Nils Teufel, Alessandra Galiè, Nelly Njiru, Eunice Kariuki, Annet Abenakyo Mulema, Isabelle Baltenweck, Chris Stephen Jones

**Affiliations:** 1 Policy Livelihoods and Institution (PIL), The International Livestock Research Institute (ILRI), Nairobi, Kenya; 2 Feeds and Forages Development, The International Livestock Research Institute (ILRI), Addis Ababa, Ethiopia; 3 Policy Livelihoods and Institution (PIL), The International Livestock Research Institute (ILRI), Addis Ababa, Ethiopia; 4 Feeds and Forages Development, The International Livestock Research Institute, Nairobi, Kenya; Indira Gandhi National Tribal University, INDIA

## Abstract

Small-scale cultivation and irrigation of planted forages can increase the availability of good-quality animal feed in smallholder farms. However, low adoption rates of improved forage technologies in most parts of sub-Saharan Africa have been observed and are partly attributed to limited understanding of gender dynamics in the context of production and utilization of planted forages. The introduction of small-scale cultivation and irrigation of planted forages is likely to interlink gender relations in the mixed crop–livestock farming system given the differences in contributions, benefits and challenges men and women farmers face. Efforts to transform livestock systems through improving adoption, scaling, and sustainability forage interventions can benefit from empirical evidence on gender relations in feed-related activities. We aim to highlight the linkage between gender relations and women’s empowerment in the production and utilization of feed resources smallholder settings. We used mixed methods, drawing on quantitative data obtained from a cross-sectional survey of 250 men and 250 women, and qualitative data obtained from eight focus group discussions in smallholder settings of the Amhara and Southern Nations, Nationalities, and People’s regions of Ethiopia, where small-scale irrigation was introduced to boost the production of cultivated forages by households. We used the Women’s Empowerment in Livestock Index tool to elicit data and analyze the empowerment of the sampled men and women. Women’s empowerment differed significantly with different dimensions of gender relations, types of forages grown, and small-scale irrigation practice. Moreover, women in households practicing small-scale irrigation of planted forages were significantly more empowered and most (80%) achieved the adequate threshold in “work balance”. The study findings point to the positive link between empowerment and the likely shifts in gender relations from the practice of small-scale production and irrigation of forages.

## Introduction

Over one billion people in low- and middle-income countries (LMICs) derive their livelihoods from livestock [1–3]. Livestock’s potential to transform livelihoods in LMICs is apparent, especially when the complex trade-offs that enable livestock’s positive impacts to be realized while minimizing and mitigating negative ones (including threats to people’s health and the environment) are well managed [4]. For instance, even though some livestock compete with people for food (grains in livestock feed), livestock contribute to the food supply by converting low-value materials, which are inedible or unpalatable for people (i.e., certain feeds such as crop residues), into milk, meat, and eggs [4]. As a store of wealth, as well as a source of income and nutritious food, livestock can provide meaningful opportunities to support livelihoods in LMICs, particularly for women livestock keepers [1], who constitute a greater proportion of the world’s poor [5]. In LMICs, it is often easier for women to acquire livestock assets (through inheritance, markets, or membership of a group) than it is for them to purchase or control other physical or financial assets, including land [6]. Although ownership of assets provides a means for women’s empowerment (by increasing their bargaining power and authority within the household and the community), the ability to make decisions on these assets and other productive resources, and to manage and make decisions on income earned through these assets, is crucial for women’s empowerment [6].

Empowerment, is a “multi-dimensional social process that helps people gain control over their own lives” [7]. It is the process of enhancing the capacity of individuals or groups to make choices and to transform those choices into desired actions and outcomes [8]. Kabeer (2012) [9] defines empowerment as the processes of change through which those who have been denied the capacity to exercise choice gain this capacity. Studies have shown that sustainable development is impossible without women’s empowerment and gender equality [7]. The empowerment of women in the livestock sector is fundamental to achieving gender equality [10], as well as improvement in livestock productivity; it is expected that households with empowered women may, in turn, have a higher propensity toward adopting new agricultural technologies and approaches [11]. Yet, as argued by Harris-Coble *et al*., (2022) [2], gender-disaggregated data related to livestock feed practices and technologies are lacking. More so, less is known about women’s empowerment and livestock.

With the rising demand for animal-source foods [12], the focus must shift to increasing livestock productivity in LMICs. Improved productivity has the potential to increase these benefits from livestock and mitigate some of the environmental risks posed by livestock, particularly for ruminant production [13]. The productivity of livestock in LMICs, however, remains low across the production systems, and the potential of the sector to support higher incomes, better nutrition [14], and progress toward gender equality is compromised. Livestock productivity is currently constrained by complex systemic challenges, and the limited supply of good-quality feed is commonly cited as one of the main bottlenecks in most African countries [15]. The low genetic potential of indigenous animal species and inadequate animal health services are factors that limit the productive capabilities and feed conversion efficiencies of animals regardless of nutritional management. However, the scarcity of high-quality and sufficient quantities of feeds is documented to be the critical constraining factor in livestock production [1, 16, 17]. This is because feeds account for up to 70% of livestock production costs and because the attainment of genetic gains or benefits from investment in livestock health hinges on good livestock nutrition [18].

In most sub-Saharan Africa production systems, good-quality forage may be available, and forage quantities may even be excess during good rainy seasons; however, availability and quality decline rapidly during the dry season [19]. The difference between availability of feed resources as dry matter (DM), metabolizable energy (ME), and crude protein (CP) and the requirements of all animal species (i.e., feed balance) shows that feed deficiency in Ethiopia is 9% for DM, whereas ME and CP deficiencies are 45% and 42%, respectively. This suggests the lack of good-quality feeds in the country [20]. Therefore, to meet the increasing demand for ASF, it is important that good-quality feed is available year-round in sufficient quantities and that the right type of animal is used for production.

Approaches to improving feed availability include the utilization of on-farm feed resources, improving the quality of by-products, or improving the ability of livestock to efficiently convert low-quality materials into high-quality nutrients for human food (thereby reducing the proportionate volume of concentrate feeds offered to livestock), and the production of planted forages through rainfed and irrigated agriculture. Small-scale irrigation is one of the agricultural activities used by rural farmers to improve overall capacities for production and income generation, and to reduce poverty [21]; this has important implications for gender relations [22]. The usual crops grown under small-scale irrigation are high-value crops, for instance vegetables (such as kale, tomatoes, and chilies), which are mainly for sale rather than domestic consumption. In irrigation literature, such crops and their enterprises are usually in the control of men and may be capital intensive. However, vegetables are traditionally placed in the women’s domain, especially where irrigation is undertaken on small plots of land with intensive care [22, 23]. Home gardening makes a positive contribution toward women’s empowerment by giving women greater control over what the household eats and control over a small source of cash income [24]. Meaningful improvements in empowerment occur when women produce beyond their household consumption and successfully leverage surplus resources to gain higher utility, and, therefore, bargaining power in their household [25]. Consequently, small-scale irrigation can be a major tool to increase women’s empowerment through greater access to and control over household income, and improvements in quality of life.

In Ethiopia, Ghana, and Tanzania, small-scale irrigation for planted forages was introduced as part of the Innovation Lab for Small-Scale Irrigation (ILSSI) project’s interventions to test the concept of cultivating planted forages (e.g., Napier grass—*pennisetum purpureum*, oat—*Avena sativa*, vetch—*vicia sativa*, desho—*pennesetum pedicellatum*, *Brachiaria sp*., and *Panicum* grasses, among others) grown under irrigation, in smoothening out livestock forage supply, focusing especially on the dry season feeding challenges [26–28]. ILSSI was a 10-year research-for-development project, implemented in two phases from 2013. The project aimed to benefit farmers by improving the effective use of scarce water supplies through interventions in small-scale irrigation. The project’s interventions involved on-farm validation of context-specific small-scale irrigation technologies through the active participation of farmers in the evaluation process. Capacity building, in terms of training and demonstrations (on alternative water lifting technologies, irrigation water scheduling, planted forage varieties, and the use of irrigated forages for dairy production), constituted an important part of the project intervention. A strategic intervention was designed to introduce the concept of cultivating improved forage varieties using small-scale irrigation to support farmers in the production of high-quality feeds for their livestock using locally available water resources (e.g., streams and boreholes) through dairy cooperatives in the Amhara and the Southern Nations, Nationalities, and People’s (SNNP) regions of Ethiopia.

Results from previous research, conducted as part of the ILSSI project from 2013 to 2018 [28], provide empirical evidence that cultivating forages improves forage availability and that, when small-scale irrigation is integrated into forage production, smallholders further increase availability of high-quality livestock feed, which in turn has the potential to improve their incomes and livelihoods. Moreover, emerging markets could provide higher returns to smallholders from sell surplus irrigated forages [28]. Given that poor rates of adoption of improved forage technologies in most parts of sub-Saharan Africa [29–31] are (partly) attributable to the fact that gender dynamics related to irrigated forage production in the region are not well understood [32], and given that the introduction of productivity-improving and labor-saving technologies are often associated to women’s empowerment, we analyzed gender relations in ILSSI project sites to appreciate the links between gender dynamics and women’s empowerment in small-scale irrigation for forage cultivation. Assessing women’s empowerment (in the context of small-scale irrigation and cultivation of forages) would help in prioritizing strategies [10] that optimize targets and the benefits from the technologies, and, thereby guarantee positive impacts for all participants.

We studied gender relation in four dimensions: gender division of labor in the production of irrigated forages; access to and control over resources; and decision-making over the production and utilization of the irrigated forages; norms and values around the dairy animal and milk ownership; and how these norms interlink to influence broad outcomes, especially women’s empowerment, in households that engage in small-scale forage production. These four dimensions were identified based on the evidence from LMICs that men make most decisions about use of productive resources while women provide much of the labor; that women have comparatively limited access to technologies and services than men do, despite the fact that technologies are necessary to enable women to perform the work [33]; that women are affected by time poverty—a result of gendered social norms that give men power over women and assign lesser value to women and their labor contributions [34]; and, finally, that women’s involvement and empowerment in decision-making related to agriculture can assist in increasing the adoption of technologies at the farm level in developing countries, for example the use of climate-smart technologies [35]. In Ethiopia, women tend to be responsible for barn cleaning, milk selling, and feeding livestock, while men take charge of sick animals and the collection and storage of forages [36, 37]. Both men and women contribute to livestock production through labor and decision-making, but they face different challenges in accessing resources and benefits from livestock production [38]. The introduction of small-scale irrigation and forage cultivation are expected to be interlinked with gender relations in the livestock–crop mixed farming systems of Ethiopia.

Our research questions, in the context of small-scale production and utilization of irrigated forages, are: (i) How do men and women differ in terms of division of labor, access to and decision-making over the production and utilization of the irrigated forages? In addition, what are the gender-based norms and values that guide their actions? (ii) Does the empowerment of women significantly differ depending on the forages they grow and whether they practice small-scale irrigation of the forages (a proxy of access to resources)? In addition, does the empowerment of women vary depending on their involvement in decisions on forage production and utilization?

**Fig 1:**
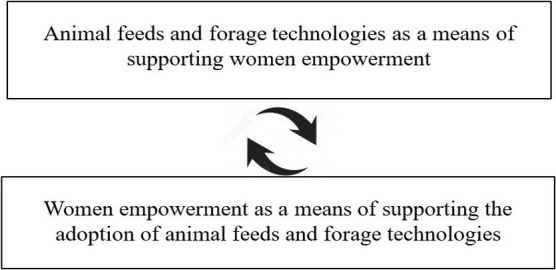
Conceptual association between empowerment and the adoption of planted forages. Source: adapted from Omondi et al. (2022) [39].

To study the relationships between empowerment, gender relations, and forage cultivation in small-scale irrigation systems, we first observe the status of four dimensions of gender relations (access to resources, division of labor, decision-making, and gender norms) among men and women in our study. Then we focus on two of these dimensions (access to resources and decision-making) in the production and utilization of forage produced under small-scale irrigation to test our hypothesis: the empowerment of women farmers is significantly related to the growing of forages, decision-making on production, utilization of planted forages, and the use of small-scale irrigation to produce planted forages (Fig 1). We used the Women’s empowerment in Livestock Index (WELI) tool to collect and assess the level of women’s empowerment. WELI has been used in several studies, either jointly or individually since the conception of the tool (see [10, 39–41]). In the next section, we present detailed description of the methods used in the study. We then report on the study findings before drawing conclusions and discussing the implications of the study.

## Materials and methods

We employed a sequential mixed-methods approach, using both qualitative and quantitative methods, to test the study hypothesis. Qualitative data collection preceded quantitative data collection and was used as an input to refine the quantitative study tool and explore gender relations. We used quantitative data to derive empowerment indices of women farmers, which were then used to compare the empowerment levels by forages planted, use of small-scale irrigation, and the levels of women’s engagement in decision-making regarding production and utilization of planted forages. The study used data from ILSSI-Ethiopia project’s 2022 face-to-face gender survey of 250 women and 250 men from 250 livestock-keeping households. The households were sampled from members of dairy cooperatives from the Amhara and SNNP regions of Ethiopia. Designed to understand the production and utilization of irrigated forages, gender relations and women’s empowerment, nutrition/dietary diversity, and perceptions about climate change and adaptation measures, the gender survey collected data between February and March 2022 from the ILSSI-Ethiopia project’s intervention districts, covering wards where ILSSI project interventions were implemented and *wards* that had dairy cooperatives but were not part of the ILSSI project intervention.

### Study site

#### Selection of study sites

**Fig 2:**
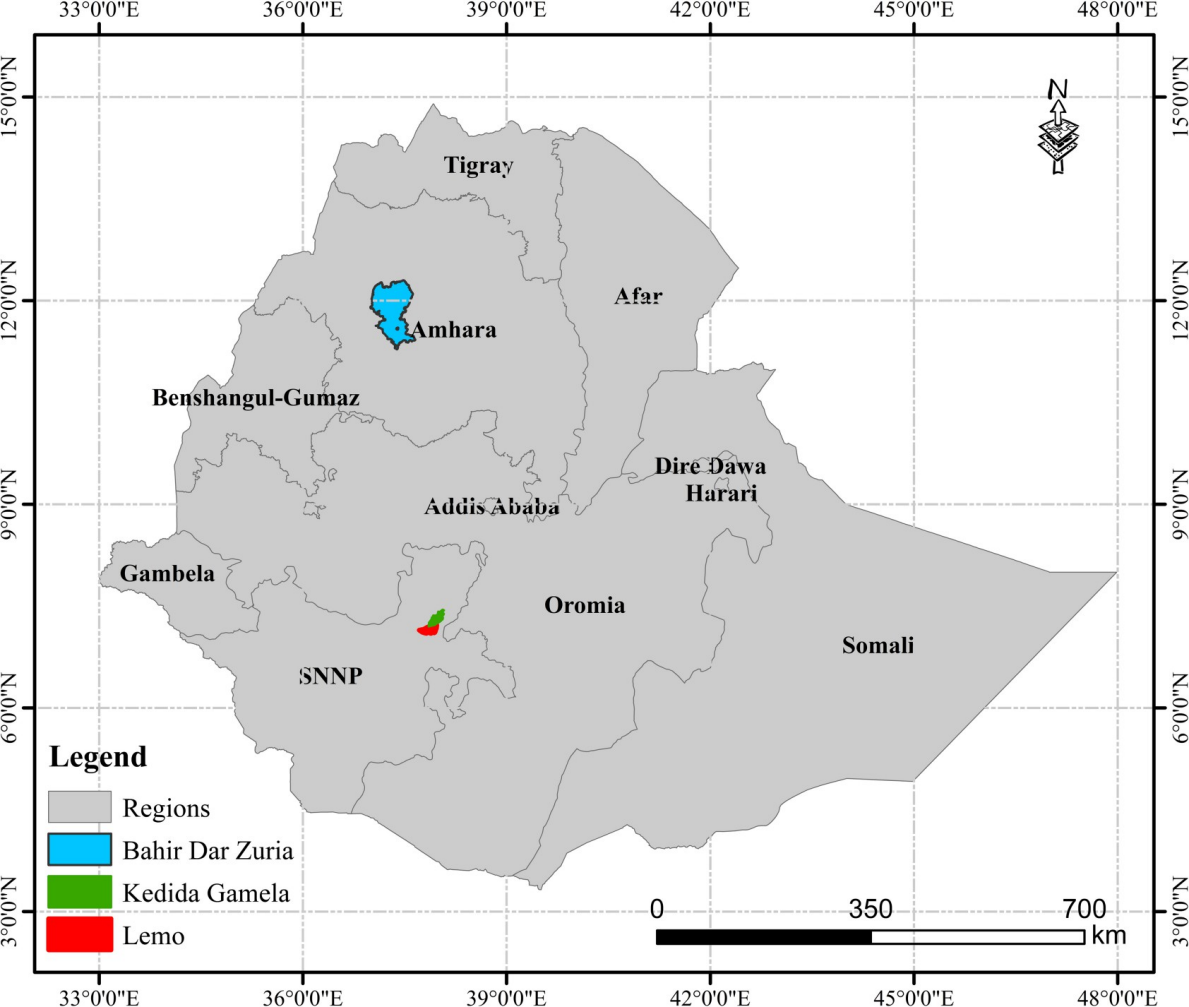
Map of study area.

Lemo and Kedida Gamela district in the SNNP region and Bahir Dar Zuria district in the Amhara region (Fig 2) were purposively selected because they have been operational sites for the ILSSI project (typically mixed crop–livestock farming areas). In Ethiopia, a district is the third level of the administrative divisions after zones and the regional states. Wards, also known as *kebeles*, are the fifth and smallest administrative unit of Ethiopia.

A stratified multistage sampling with three hierarchical stages was used as a sampling strategy for conducting the quantitative WELI survey. The first step was to identify intervention dairy cooperatives (where ILSSI project intervention activities were implemented) and non-intervention ones (where no ILSSI project intervention activities were implemented) in the selected *districts* and, consequently, *wards*. The cooperatives were the sampling reference, as there is only one dairy cooperative in a *ward*. A stratified random sampling approach was used to select two *wards*, one from a list of intervention *ward* and one from a list of non-intervention *wards*, as the second step. The cooperative members received training sessions, demonstrations, and technical support on the production and utilization of irrigated forage through the ILSSI project. Six cooperatives (two per district) were sampled from the three districts. The third step involved randomly selecting members of cooperatives from intervention and non-intervention *wards*.

#### Description of the study sites

Bahir Dar Zuria is a district in north-western Ethiopia, located in the West Gojam zone of Amhara National Regional State [42], covering a total area of more than 151,000 hectares. Lemo and Kedida Gamela districts are located adjacent to each other in the SNNP region. Lemo is one of the most densely populated areas in the country, with an average land holding per household of about 0.5 hectares (the district covers an area of 38,140 hectares). It is characterized by rugged highland and hilly areas, including a mountain range with steep slopes, and lies in the “Woina Dega” agro-climatic zone [43]. In contrast, Kedida Gamela has a highly undulating landscape with three contrasting agro-ecologies [“Kolla” (arid and semiarid), “Woina Dega” (semi humid), and “Dega” (cool and humid)] depending on the altitude and is about two-thirds the size of Lemo (the district covers an area of 10,890 hectares [44]). Lemo and Kedida Gamella districts receive bimodal rainfall (the short rainy season occurring during the months of February and March and the main rainy season from June to September)—the average annual rainfall is about 1,100 mm (slightly higher in Kedida Gamella, 1,100–1,400 mm), whereas Bahir Dar Zuria receives an annual rainfall of 1,000 mm.

The farming system in the three study sites is mixed crop–livestock farming. Livestock reared include cattle, sheep, goats, equine, and poultry. The cattle population is mainly the local type. However, in the peri-urban areas, farmers (particularly those in Bahir Dar Zuria) have crossbred (improved) cows, hence produce and supply milk to the local market through cooperative organizations. Cattle play a key role in the system as a source of draft power, milk, and meat. Dairy production is supported by farmer cooperatives. The main source of water for irrigation is shallow wells in Bahir Dar Zuria. Ponds, shallow wells, and streams/rivers are the source of water for farmers who practice small-scale irrigation in Lemo and Kedida Gamella. Irrigated forage is a recent development initiative introduced through the Africa RISING and ILSSI projects [26, 27, 45].

### Study design, sampling, and analysis

#### Research ethics

The study was approved by the International Livestock Research Institute—ILRI Research Ethics Committee (ref. ILRI-IREC2020-45, with amendment ref. ILRI-IREC2020-45/1). The process of obtaining informed consent from FGD participants involved setups to protect participants’ privacy, keep information confidential, and allow the participant to remain anonymous despite collecting individual participants’ personal and farm characteristics such as age, marital status, land allocated to the forages, and land under forage irrigation. While the participants were informed of their right to withdraw from the study at any time none of the participants chose to withdraw from the study. Household survey participants signed both written consent (translated into Amharic language) and a digital version of the consent on the Open Data Kit tool that was used to gather data during the computer-aided personal interviews (CAPI). Participants retained a copy of the signed (by both the respondent and the interviewer) written consent form. To ensure confidentiality and anonymity of the study participants, only the research team handled the collected data.

#### Inclusivity in global research

Additional information regarding the ethical, cultural, and scientific considerations specific to inclusivity in global research is included in the Supporting Information (S1 Checklist).

#### Qualitative survey: Sampling, and data analysis

We used a phenomenological study approach where the inquirer then collects data from persons who have experienced a phenomenon of interest and develops a composite description of the essence of the experience for all of the individuals [46]. The qualitative survey was conducted at the ward level. Four wards were randomly selected, with two representing those that had a dairy cooperative (one in the SNNP region and one in the Amhara region) where the ILSSI project interventions were promoted. The other two selected wards were from those that had dairy cooperatives where no ILSSI project interventions were promoted (one in the SNNP region and one in the Amhara region). Qualitative data were gathered through the FGDs. The criteria for selection of the FGD participants (farmers who were members of the cooperatives) were based on the level of adoption of the irrigated forage production and utilization practices. The sample comprised (i) men and women who practiced both the cultivation of forages and the application of small-scale irrigation; (ii) men and women who practiced only the cultivation of forages; and (iii) men and women who neither planted forages nor practiced irrigation, even though they knew about the technologies. For the non-intervention cooperative, the FGD participants’ selection criterion was just membership of the cooperative. Table 1 summarizes the FGDs implemented in the two regions.

**Table 1:** Sample size for the quantitative survey and qualitative survey FGDs.

Region	District	Ward	ILSSI project intervention area	Quantitative survey	Qualitative survey	
				Number (*n*) of households	Type of informants	Number (*n*) of FGDs
Amhara	Bahir Dar Zuria	Sebatamit	Non-intervention	74	–	0
		Robit Bata	Intervention	90	1. Small-scale irrigated forage farmers	2^
					2. Small-scale irrigated forage farmers	2^
SNNP	Lemo	Haise	Intervention	76	1. Small-scale irrigated forage farmers	2^
					2. Small-scale irrigated forage farmers	2^
		Ambicho Gode	Non-intervention	114	–	0
		Detcho Demala	Non-intervention		1. Small-scale irrigated forage farmers	2^
	Kedida Gamela	Zeto Shedera	Intervention	84	–	0
		Jore	Non-intervention	62	–	0
Total number from intervention area				250		8
Total number from non-intervention area				250		4

Note: FGD, focus group discussion; ILSSI, Innovation Laboratory for Small-Scale Irrigation.

^One comprising female farmers only and one comprising male farmers only.

In last column, 0 indicates that no FGD was planned/conducted in the ward.

In each ward, a women’s FGD and a men’s FGD was conducted concurrently, with 8–10 participants per FGD, totaling 12 FGDs (six women’s FGDs and six men’s FGDs). It is worth noting that farmers in the study areas self-identify with the historical understanding of sex, which “focuses on the biological and physiological determinants of male or female identity” [47]. Consequently, the notion of a sexual binary, in which one simply “is” male or female, was not imposed on survey respondents but was adopted as usual practice in the community (in other words, we conformed to the community’s usual presentation of gender identity as binary–male and female—and did not extend to explore other categorization of sexual identity or sexual-orientation). The FGDs were conducted separately for men and women to create a conducive environment for men and women participants to openly express their opinions and share their experiences, and to enabled comparison between men’s and women’s perceptions of empowerment and the role of gender in the adoption of irrigated forage production. Qualitative data with emphasis on people’s ‘lived experiences’ are suitable in locating the meanings people place on their perceptions, assumptions and presuppositions, and connecting these meanings to the social world around them [48, 49]. Consequently, the qualitative survey was intended to provide insights into the status of gender relations and factors influencing the uptake of the planted forages an/or the small-scale irrigation practices, and how the community understood the term “empowerment” in its local context. The field transcripts were translated from Amharic to English. We used open coding in NVivo; QSR International, Warrington, UK) to develop a codebook of deductive themes based on the study’s research questions and identify new themes that emerged from the interviews. Decisions on which emerging themes were discussed by the researchers and emerging insights informed the new codes that meaningfully contributed to the analysis. The data were then synthesized by theme and interpreted as discussed in this paper.

#### Quantitative surveys: Sample size estimation, sampling, and data analysis

A survey tool combining the WELI and technical questions on small-scale irrigation and forage production and utilization was designed and tested. The estimation of the required sample size for the quantitative household survey was based on power calculations using data from a previous WELI survey conducted in Tanzania [10]. The sampling unit was a farm household, while the unit of analysis was the individual farmer from within a farm household. The sample size estimation yielded a minimum required total sample of 250 households. The study’s design involved collecting data from the 250 households in which two primary decision-makers, a woman (“index woman”) and her husband, or the main adult male, were to be interviewed, yielding 500 individual responses.

A sampling frame of all dairy cooperative member households in the selected administrative areas (intervention and non-intervention wards) was obtained from the respective administration offices. Because the presence of both an index woman and an adult man in a household was a requirement, cooperative member households that did not fulfill this criterion were excluded from the sampling frame. Based on the power calculations without further clustering (to improve sampling efficiency), 125 households were selected from the intervention wards. The same number of households was also selected from the non-intervention wards, resulting in a total of 250 sampled households (Table 1).

A cross-sectional survey was conducted to obtain quantitative data through face-to-face interviews with the index women and an adult man in each sampled household. The woman and man from each household were interviewed separately, using the same structured questionnaire. The purpose of including men was to estimate the Gender Parity Index [the difference between the men’s empowerment and women’s empowerment indices] in a bid to explore patterns in gender relations within the surveyed households. The WELI-related variables from the data are aggregated into 13 indicators, from which empowerment index values are calculated. Each indicator is derived from data obtained from individual men and women respondents from households that derive a considerable part of their livelihood from livestock production. S1 Table provides a detailed description of the indicators, the definitions, and adequacy thresholds for each indicator used to calculate the final index (WELI) score.

The method of scoring used is analogous to standard methods for computing other variants of empowerment indices [39]. To determine whether an individual has achieved a minimum level of empowerment for a particular indicator, responses to questions contributing to an indicator are considered together, sometimes in several steps. Each respondent is classified as either adequate (= 1) or inadequate (= 0) for a given indicator by comparing their responses with the survey questions with a given threshold (S1 Table). A respondent’s empowerment score is simply the weighted average of their adequacy scores for the 13 indicators (all weighted 1/13). If her/his score is greater than 74%, (i.e. being adequate in at least 10 out of 13 indicators), then s/he is considered empowered [39].

To test the significance of the relationship between women’s empowerment and gender relations dimensions, we used descriptive statistics (mainly frequencies, and means from quantitative data, supported by qualitative findings) to describe the characteristics of farms and farmers in the study area and, dimensions of gender relations in the context of the production and utilization of planted forages. Furthermore, we applied hypothesis testing (independent-sample tests of means) to assess whether empowerment significantly relates to different dimensions of gender relation scores. We test whether the empowerment score of any two populations represented by the sample are statistically different, as illustrated by Eqs 1 and 2. We first compute the empowerment score (the WELI) and conduct a two-sample test of means by sex of the geographical location of the respondents (two regions in Ethiopia), types of forages grown, whether or not irrigation is practiced, women’s participation in decision-making on irrigated forages, and the utilization of forages.

$$H_{0}:\mu_{group1}=\mu_{group2}$$

$$H_{A}:\mu_{group1}\neq \mu_{group2}$$ (1)

where *μ*_*group*1_ represents the mean for the first group for the null H_0_, and the alternative, H_A_, hypotheses.

To decide between *H*_0_ and *H*_A_, we compared the *t*-statistic (*Eq 2*) to the *t*-distribution with (*n* − 1) degrees of freedom (df), where *n* is the sample size for the group and *s* is the standard error:

$$t=\frac{\mu_{group2}-\mu_{group1}}{\sqrt{\left(\frac{s_{group1}^{2}}{n_{group1}}+\frac{s_{group2}^{2}}{n_{group2}}\right)}}$$ (2)

## Results

In this section, we present our findings from the analysis of the data obtained from the study sample (both qualitative and quantitative). We start by presenting the demographic characteristics of our respondents, including the forages they cultivate and whether/how irrigation is practiced (a proxy of the access to resources dimension of gender relations). We then present observations from data analyses of the other three dimensions of gender relations (from both qualitative and qualitative data), focusing on the division of labor, gender norms, and decision-making on the production and utilization of planted forages. Finally, we present the results of our empowerment analyses, highlighting observed differences in relation to the sex of the respondent, decision-making on the production and utilization of planted forages, the forages cultivated, and practicing small-scale irrigation.

### Selected social demographic characteristics of the sampled households focusing on the dimensions of gender relations

Each of the sampled households had, on average, four adult members with on average two adult women. The households also kept livestock, with most households keeping more than one livestock species. On average, the households were found to be keeping approximately four large ruminants (mainly dual-purpose cattle, including milking cows and oxen), eight chickens, and three small ruminants (sheep and goats). Additional social demographic characteristics are presented in S2 Table, disaggregated by decision-makers in the households.

The average age of the index women was 38.3 years (slightly lower in the Amhara region, at 37.6 years, than in the SNNP region, at 40.9 years). The main male in the households (spouses) were, on average 7 years older than the index women in both regions and more of them were educated compared to the women. For instance, only 17.7% of spouses did not have any schooling, compared with 37.6% of women. The proportion of index women who lacked formal schooling was higher in the Amhara region (62.2%) than in the SNNP region (23.9%). Majority (96%) of the index women were mostly present at home, compared to approximately 74% of the main males.

#### Production and utilization of forages

The average area of land cultivated in the study was approximately 0.94ha. However, farmers from the Amhara region cultivated significantly (*t* = 2.25, df = 248; *p* < 0.05) more land (1.08ha) than their SNNP counterparts (0.88ha). Most of the households (68%, 72%, and 70% in the Amhara region, the SNNP region, and the entire sample, respectively) cultivated forages on between 0.25 and 1.25ha. Farmers cultivating about 0.25ha allocated 20% of their land to growing forage, whereas farmers cultivating more than 1.25ha allocated less than 10% of the cultivated land to growing forage.

Approximately 66% (331 out of 500 respondents) produced forage, with the practice being more popular in SNNP households (76%, compared with 48% of households in the Amhara region). None of the 34% of the households that did not produce forages used irrigation (Table 2). Among the forage producers, 18% used irrigation for forage cultivation, with more households in the Amhara region using irrigation (28%, compared with 16% of households in the SNNP region). Some households irrigated manually using buckets, hosepipes, or watering cans, at least once a week, whereas others did it biweekly. Surface irrigation and the use of sprinklers were not common. Most irrigating households used buckets or hosepipes to obtain water from groundwater sources. Rivers and ponds were rare sources of irrigation water.

**Table 2:** Number of households practicing and not practicing irrigation.

Category of households	Irrigating (*n*)	Not irrigating (*n*)	Total number of respondents (*n*)
Cultivating forages	59	272	331
No forages	0	169	169
Total number of respondents	59	441	500

Napier and desho grasses were the main forages, grown by over 66% of the sampled farmers (Table 3). These were followed by oats, alfalfa, vetch, *Sesbania*, *Desmodium*, and cowpea, which were grown by between 4% and 20% of farmers. *Panicum*, pigeon pea, and fodder beet were grown by less than 1% of farmers. Overall, the most produced forage in both the SNNP and Amhara regions was Napier grass (grown by 75% of the households growing forages), followed closely by desho (grown by 66% of the households). Desho was mainly grown by farmers in the SNNP region, whereas Napier grass was more common in the Amhara region (Table 3) but also grown by about 70% of farmers in the SNNP region. Moreover, most households (55%) grew more than one forage type.

**Table 3:** Frequency (%) of households growing and preferences for different forage types.

Percentage number of respondents (index woman responses) reporting growing forage type				Percentage of respondents (index woman responses) reporting “preferring” a forage type			
Forage type	Amhara (*n* = 39)	SNNP (*n* = 127)	All (*n* = 166)		Amhara (*n* = 39)	SNNP (*n* = 127)	All (*n* = 331)
Napier	92.31	69.29	74.70		92.31	29.13	41.99
Desho	0.00	86.61	66.27		0	56.69	47.43
Oats	0.00	11.81	9.04		0	7.09	4.23
Alfalfa	0.00	9.45	7.23	}	7.69	7.09	6.35
Vetch	0.00	3.94	3.01				
Cowpea	2.56	4.72	4.22				
*Desmodium*	0.00	20.47	15.66				
*Sesbania*	7.69	4.72	5.42				
*Panicum*	0.00	0.79	0.60				
Pigeon pea	0.00	0.79	0.60				
Fodder beet	0.00	0.79	0.60				

The results presented in Table 4 demonstrate that the most popular pattern of forage production, indicated to be grown by 41% (136 out of 331) of the respondents, was desho and Napier grasses grown together (with or without other forages). By comparison, 31% of farmers were growing Napier grass without desho grass (with or without other forages) and 26% were growing desho grass without Napier grass (with or without other forages). Most forages were grown in an outfield (a field outside the homestead), mostly less than 1 km from the homestead. Some households produced forage in a field within the homestead.

**Table 4:** Frequency of responses from men and women on the combinations of forage types grown in their households.

Forage combinations grown	Value type	Respondent category and sex		
		Husband/main male adult in the household	Index woman	Total
Napier grass (only or grown together with other forages—excluding desho grass)	Number	50	51	101
	Frequency (%)	30.30	30.72	30.51
Desho grass (only or grown together with other forages—excluding Napier grass)	Number	42	43	85
	Frequency (%)	25.45	25.90	25.68
Desho and Napier grasses grown together (only or with other forages)	Number	68	68	136
	Frequency (%)	41.21	40.96	41.09
Other forages (not grown together with Napier or desho grass)	Number	5	4	9
	Frequency (%)	3.03	2.41	2.72
Total	Number	165	166	331
	Frequency (%)	100.00	100.00	100.00

**Fig 3:**
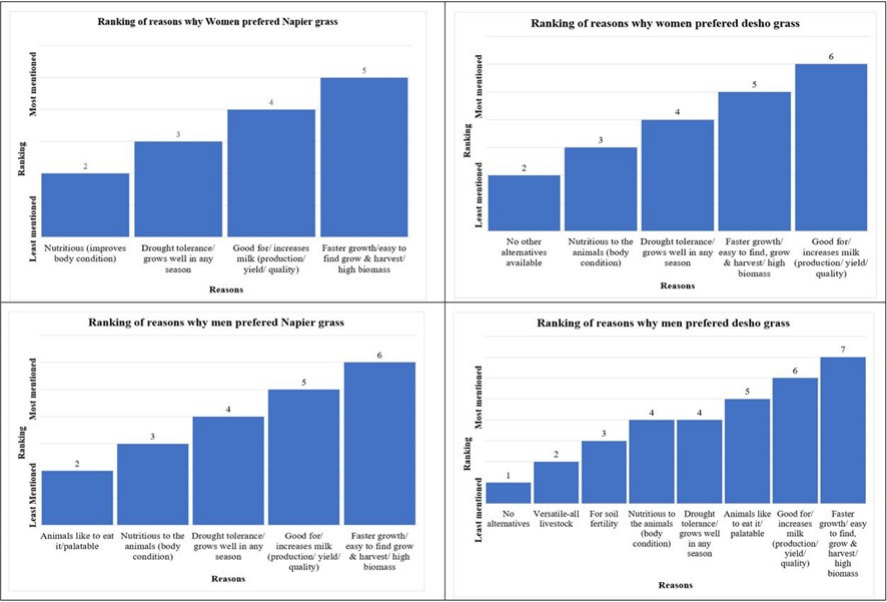
Reasons for preferring desho and Napier grass by men and women.

Regarding the most preferred forage in the study area. The results (Table 3) reveal that most respondents (regardless of their sex) preferred desho grass (47%) or Napier grass (42%). The preferred forage type however, differed by region (in Amhara, nearly all respondents (92%) preferred Napier grass, whereas in the SNNP region only about 30% preferred Napier grass). The three reasons most cited by women and men farmers for preferring Napier and desho grasses were, in order, (i) faster growth, (ii) increased milk production, and (iii) drought resistance (Fig 3).

Participants indicated the main sources of feed for ruminant livestock in the household. We gauged the importance of forage type by the ranking (order of mentioning), denoting the significance the respondents placed on the forage type compared with other available alternatives. Since most respondents indicated growing more than one forage type, we considered the frequency of a forage type being mentioned as an indication of the importance of the forage type to the respondents. Approximately 25% (13.9% men and 11.5% women) of the forage type mentions was crop residues, indicating that it was the most important feed for ruminant livestock. Crop residues come from crops such as wheat, teff, and maize, as well as legume crops. Seventeen percent (7.6% men and 9.7% women) of respondents mentioned purchased feed (concentrates) by. Cultivated forages were the third most important feed, mentioned by 17% (7.6% men and 9.0% women) of the respondents, ahead of grazing pastures (mentioned by 15.5% of the respondents—7.6% men and 7.9% women). About 11.3% (4.2% men and 7.1% women) mentioned that they scavenge for tree leaves, shrubs, or weeds as feed for their ruminant livestock. It is interesting to note that slightly more women than men depended on cultivated forages and purchased concentrates.

About 9.8% of women and 11.6% of men respondents mentioned that they purchased either green forage or crop residues for their ruminant livestock. Most of these households indicated that they had to travel between 1 and 5 km to purchase the forages or crop residues, whereas a few had to travel to locations that were 5–10 km away.

**Fig 4:**
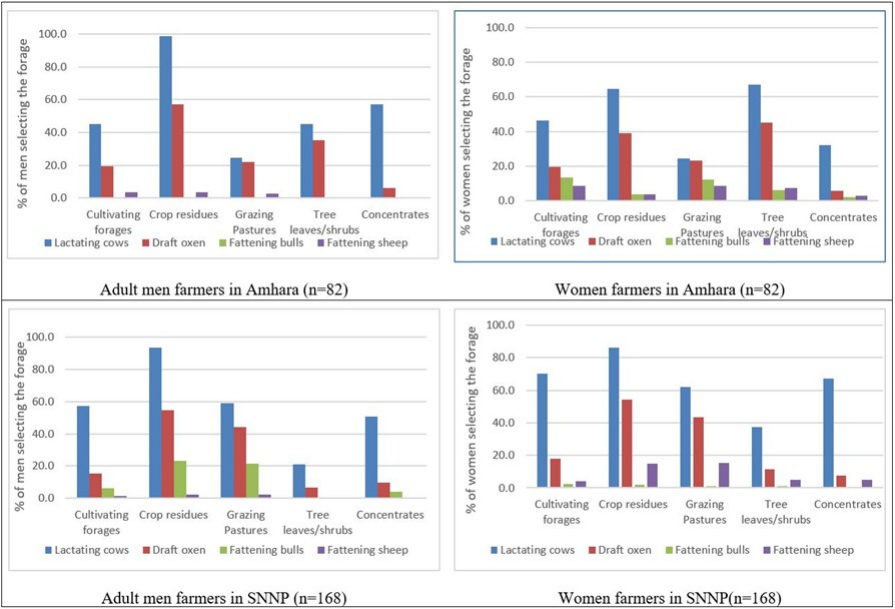
Priority feed resources by men and women for different livestock types.

Regarding the livestock species they fed the feed resources, our results revealed that lactating cows were prioritized for feeding from all the feed sources by both men and women in both the SNNP and Amhara regions (Fig 4). The second category of animals to be prioritized for feeding was draft oxen. However, the cultivated forages were clearly more important for the lactating cows than for the draft oxen, which were mostly fed on crop residues or grazed in open pastures. Fattening bulls and fattening sheep were not highly prioritized. Scavenging of tree leaves, shrubs, and weeds for lactating cows and draft oxen was mentioned by both the adult men and the index women. Men in the SNNP region did not mention scavenging tree leaves, shrubs, and weeds as being an important source of feed for fattening bulls or the fattening sheep. Only a small percentage of respondents in the SNNP region said that they used leaves, shrubs, and weeds as feed for fattening bulls and sheep. In the Amhara region, male respondents did not mention feeding forages to bulls, but the women indicated using all the different sources for feeding bulls, particularly those kept for fattening (beef).

The sale of different forages was not common among the respondents of the survey. Only three farmers indicated selling forage from their farms. They sold the forages from their farm gate. For most households, their cattle herds were so big that they needed all the forage that they produced.

#### Gender division of labor in the forage cultivation, irrigation of forages, and feeding of livestock

There is a saying “sete wede majet wend wede adebabay,” which means “women for the home and men for the public”, and it influences the way tasks and responsibilities are shared in a typical rural Ethiopian household. Women took responsibility for roles in the homestead, that is, productive and reproductive, and they are rarely encouraged to participate in public spaces. Feeding various livestock species was considered a woman’s role, except when the animals are out in the grazing fields, a task often executed by the men. In some circumstances, grazing vulnerable animals (the sick, young, and old) is also a woman’s role. Female members of the households also cultivated and irrigated forages as well as feeding the animals with the forages.

Data collected using the WELI tool, capturing the 24-hour records of activities performed by each respondent, revealed that women spent significantly more time working (in general) than men. Although there was no significant difference in the total time men and women spent in raising livestock (large ruminants, small ruminants, and poultry/other small livestock), considering that women mostly double up such duties with childcare, in general, women spent approximately 1 hour more than men working in a day (i.e., 47.76 min, on average; *t* = –3.61, df = 498; *p <* 0.01). The community indicated that there is some change in culture, for instance, it is now becoming common to see men do chores that traditionally were exclusively designated for women. A woman from the SNNP FGD mentioned:

“… Especially in times of crisis men step in to help their wives and the number of men who help their wives is increasing. This change has come as the government advocates for gender equality. They have been teaching us for so long.”

#### Norms and values around dairy animals and milk

We observed from the qualitative study that women access, process, and market milk products and use the money generated for the household. The norms, especially in the SNNP region, protect this right by shaming men who attempt to take away the control of milk from women. Such men are called *Qonqoracho womenish*, which translates as “stingy” or “selfish.” Ownership and control of milk, milk products, and the income generated from them is a valuable resource in the hands of the women. and it has implications for women’s own agency and for their households’ food security, nutrition, and well-being. Moreover, the dairy cooperatives in the SNNP study area were led by women. In the Amhara region, milk resource is not culturally secured in the hands of women; men generally own the cow as well as the milk. Women in the Amhara region play a large part in selling milk/milk products (94.4%), but have less of a say in the final decision about how the money generated from the sale is used (only 47.8% reported having a role in this) [50]. The cooperatives in the Amhara region were led by men.

#### Decision-making on cultivation, irrigation, and utilization of forages

The study captured up to three decision-makers for all the activities investigated. The three decision-makers were mostly the index woman, and the spouse, and, in some cases, other family members (mostly a son or a daughter). The index woman and the spouse participated (either solely or jointly with others) in approximately 90% of decision-making on the cultivation and irrigation of forages. The children who participated in decision-making had an average mean age of 22.4 years. Most of these children (62.7%) indicated that they were able to carry out a full adult workload.

Regarding decision making about which livestock species are feed from the feed resources available to the households, respondents from both the Amhara and SNNP regions indicate that the index women were the main decision-makers about which animals would be fed on which feed resources. In 86% of the responses, index women were mentioned to be the primary decision-makers on feeds and the livestock species that would be fed on them (i.e., ranked based on a woman being mentioned sole decision maker or a joint decision maker–either being mentioned first or second). The results in Table 5 reveal that women had greater levels of participation in decisions regarding the production of irrigated forages than men and were the predominant decision-makers on utilization of forages.

**Table 5:** Frequency of participation (%) in decision-making on the production and utilization of irrigated forages.

Respondent category and sex		Level of participation (%) in decision-making regarding production of irrigated forages		Predominant decision-maker on utilization of forages	
		Zero to low	High level	Male	Female
Husband/main male adult in the household	Male (*n* = 250)	36.00	64.00	14.00	86.00
Index woman	Female (*n* = 250)	37.00	63.00	17.00	83.00

### The nexus between empowerment and the production and utilization of irrigated forages

#### Local meanings of empowerment

In sex disaggregated FGDs, men and women informants were asked what empowerment meant to them and whether there were local terms or terminologies that were used to describe the concept of empowerment. From the FGDs, we found that there was no general meaning of empowerment, that it was contextual and depended on several factors, for instance the sex of the persons describing empowerment and the community’s situation. For example, we observed that the term *ebulla* (“peace mediators”) was used to describe empowerment. Terms with the meaning of strength and hard work, i.e., a strong hardworking man or woman, emerged as one of the most common descriptors of empowerment, used by both men and women, to define both an empowered man and an empowered woman. We also observed differences between descriptors used by men and women, particularly those that border knowledge. Intelligence, leadership, and educated are words that were mostly used to describe men. We observed that there are two ways in which someone could be considered “empowered” in the community, through inheritance (being born into an empowered family) or by working hard (earning it). Based on the local definitions, an empowered family could be one whose members are cultural leaders, have invested in education over the years, and play roles in mediating peace deals in the community. There is a high probability that anyone born into such a family will play such roles. The terms used for this were *blata* in the Amhara region and *azematche* in the SNNP region. When a woman marries the *azematche* (or *blata*), she gains “empowered status” through her husband, called *ette gisste*.

From both the men’s and women’s perspective, in both the Amhara and the SNNP regions, women’s empowerment is linked to a kind of community scorecard of performance relating to “care work” for the household and the community. Taking diligent care of their husband and the children, guiding the family, and managing domestic chores ranked highly. It emerged that women’s capacity to balance their spending with income earned from their farm and not always expecting the husband to provide all she required was considered being empowered. Not borrowing or expecting support from the neighbors (e.g., in the provision of food for the household, school fees, and other needs) was also considered empowerment for the women. Being able to engage in public speaking and expressing one’s ideas and opinions boldly were considered a “sign of empowerment” for women, with such a woman being called a *gobez*. Some considered the empowered women to be like “a man/manly/masculine”, Although this was considered empowerment, the term is derogatory in some instances but indicates respect in other instances; for example, a widowed woman who is able to protect and provide for her family, and who participates in public affairs, is respected, whereas a married woman who does the same may be referred to as *gobez* in a belittling way, to express disapproval of her defiance of society’s expectation that she will not be vocal and to indicate that decision-making is a male domain. The men associated women’s empowerment with the respect of a woman for her husband and the traditional household duties of a woman, that is, childcare, nurturing, and providing labor on the farm as well as saving seeds.

Men in an FGD in the Amhara region linked women’s empowerment with women’s ability to fetch and ferry water from the river using donkeys. Furthermore, it was interesting that, from men’s perspective, having enough fodder for livestock was a vital component of empowerment for women. Women cultivating and conserving feeds for the dry season was considered a characteristic of empowerment and of a good wife:

*… a woman who has compost*, *she prepares organizes*, *stores*, *and manages animal feed in good manner* (a respondent, Amhara men’s focus group discussion).

Others (men) viewed an empowered woman as a peace maker and mediator when there were conflicts among neighbors. An empowered woman was considered diligent (*tatari*), and able to push and motivate her lazy spouse (husband). In contrast, one man from the Amhara region believed that women are subordinate to men, and their empowerment is based only on how they handle domestic chores and not on their role in public affairs. For both men and women, the concept of learning and knowledge was considered a sign of empowerment. Men and women who adapted innovative technologies by apply them and “taught or advised” the rest of the community, inspiring them with knowledge and experiences, were considered empowered. Men always mentioned that “when a woman was performing as well as the men in production activities, then such a woman was considered empowered.” Table 6 presents a summary of local words describing “empowerment”, by the described (a man or a woman) and the describer (men or women).

**Table 6:** Local description of empowerment, by who describes and who is described.

Local words describing “empowerment”	Described (who)		Describer (by who)	
	A man	A woman	Men	Women
*Gobez serategna*—stronger worker	✓	✓	✓	✓
*Gobez*—hard worker, public figure	✓	✓	✓	✓
*Lehizbum genbar qedem*’—an example	✓	✓	✓	✓
*Qoutero gonticho*—strong man	✓		✓	✓
*Jegina/yejeguna jegina*—a hero/superhero	✓		✓	✓
*Negus*—a king, strong man	✓		✓	
*Blata—*influential man	✓		✓	
*Azematche—*crowned leader	✓		✓	
*Ebulla*—mediator, fair, leader	✓		✓	
*Habtam*—rich, benevolent man	✓			✓
*Yesun ateqeto gorbetochun yelew*—change agent	✓			✓
*Lebam*—intelligent and a doer	✓			✓
*Mekari*—advisor	✓			✓
*Abageda*—lead farmer	✓		✓	✓
*Hetemare*—educated and rich	✓		✓	✓
*Sete net alet—*hard-working woman		✓	✓	
*Qoutera totelette*/q*outeretti*/g*obez sete*’—strong women		✓	✓	✓
*Sete adari*—role model		✓	✓	
*Denna konjo*—exceptionally beautiful woman		✓	✓	
*Tiiru yeset woizero*—feminine woman		✓	✓	
*Gisste*—wife of the a*zematche*		✓	✓	
*Tatari*—diligent		✓		✓
*Teru Sete—*good woman		✓		✓

#### Empowerment scores among the study respondents

The results of the calculation of the WELI score (Table 7) reveal that women from the sampled households were significantly less empowered (i.e., had slightly lower 3DE score) than their male counterparts, and that a lower percentage of women than of men achieved empowerment. The results for the individual indicators, presented in Table 8, reveal that both men and women were found to be achieving low adequacies on the “autonomy in income” and “physical mobility” (“visiting important locations”) indicators. Moreover, women were at par with men on two indicators (i.e., an equal number of men and women achieved adequacy): “input in productive decisions (agriculture)” and “ownership of land and other assets.” More women than men attained adequacy on three indicators: “productive decisions (livestock),” “group membership”, and “membership in influential groups”.

**Table 7:** The WELI scores by sex and decision-making on irrigated forage production and utilization of forages.

Indicators	Sex of the respondent			Level of women’s participation in decision-making on irrigated forage production			The predominant decision-maker on the utilization of forages		
	Women	Men	*t*-test	Zero to low	High	*t*-test	Men	Women	*t*-test
Number of observations	250	250		92	158		38	212	
3DE score	0.87 *(0*.*01)*	0.91 *(0*.*01)*	*t* = 2.83 df = 498 *p* = 0.01**	0.82 *(0*.*02)*	0.89 *(0*.*01)*	*t* = 3.48 df = 248 *p* = 0.02**	0.86 *(0*.*03)*	0.87 *(0*.*01)*	*t* = 0.37 df = 248 *p* = 0.03**
Disempowerment score (1 – 3DE)	0.13 *(0*.*01)*	0.09 *(0*.*01)*		0.18 (*0*.*02)*	0.11 *(0*.*01)*		0.14 *(0*.*03)*	0.13 *(0*.*01)*	
% achieving empowerment	63.20	74.00		51.09	70.25		63.16	63.21	
% not achieving empowerment	36.80	26.00		48.91	29.75		36.84	36.79	
Mean 3DE score for not yet empowered	0.64 *(0*.*01)*	0.64 *(0*.*01)*		0.63 *(0*.*01)*	0.64 *(0*.*01)*		0.63 *(0*.*02)*	0.64 *(0*.*01)*	
Mean disempowerment score (1 – 3DE)	0.36 *(0*.*01)*	0.36 *(0*.*01)*		0.37 *0*.*01)*	0.36 *(0*.*01)*		0.37 *(0*.*02)*	0.36 *(0*.*01)*	
Gender Parity Index (GPI)	0.95 *(0*.*01)*			0.93 *0*.*01)*	0.97 *(0*.*01)*		0.88 *(0*.*12)*	0.95 *(0*.*01)*	
% achieving gender parity	69.60			56.41	78.08		75.00	68.93	
% not achieving gender parity	30.40			43.59	21.92		25.00	31.07	
Average empowerment gap	0.15 *(0*.*01)*			0.16 *0*.*02)*	0.14 *(0*.*02)*		0.49	0.15	
Women’s Empowerment in Livestock Index (WELI) score	0.88 *(0*.*01)*			**0.83** *(0*.*02)*	**0.90** *(0*.*01)*		**0.87** *(0*.*15)*	**0.88** *(0*.*02)*	

Robust standard errors of individual-level estimates are indicated in parenthesis.

** indicate level of significance at 5%.

**Table 8:** Percentage of respondents achieving adequacy in specific indicators, by sex and decision-making on irrigated forages.

Adequacy (%) by indicators	Sex of the respondent			Decision-making on irrigated forages			
	Women (*n* = 250)	Men (*n* = 250)	All (*n* = 500)	Level of women’s participation in decision-making on irrigated forage production		The predominant decision-maker on the utilization of forages	
				Zero to low (*n* = 92)	High (*n* = 158)	Male (*n* = 38)	Female (*n* = 212)
Autonomy in income	40.6	46.6	43.1	38.04	40.51	34.21	41.04
Self-efficacy	65.0	85.7	75.38	66.30	67.09	47.37	68.40
Attitudes toward domestic violence	75.9	80.5	78.2	83.70	70.25	68.42	75.47
Input in productive decisions—agriculture	97.4	97.4	97.4	96.74	97.47	97.37	97.17
Input in productive decisions—livestock	94.7	93.2	93.9	95.65	93.67	97.47	94.34
Ownership of land and other assets	100.0	100.0	100.0	100.00	100.00	100.00	100.00
Access to and decisions on credit	75.9	90.1	83.3	82.61	73.42	84.21	78.77
Control over the use of income	78.6	82.3	80.5	69.57	83.54	92.11	78.30
Work balance	33.6	76.7	70.3	58.70	70.89	57.89	66.51
Visiting important locations	33.8	51.5	42.7	35.87	34.81	36.84	35.38
Group membership	99.6	96.2	97.9	98.91	100.00	97.37	100.00
Membership in influential groups	86.5	77.8	82.14	77.17	90.51	89.47	86.32
Respect among household members	72.9	71.8	72.4	54.35	82.28	84.21	72.17

More men than women attained adequacy in “work balance”, “autonomy in income”, “physical mobility” (“visiting important locations”), and “respect among household members”, which were the indicators that contributed highly to the disempowerment of all respondents in general. “Autonomy in income” was more pronounced as a contributor to the disempowerment of women than of men, whereas “respect among household members” was more pronounced for men. Unlike men, “work balance” was one of the top five contributors to the disempowerment of women (Table 8). Additional empowerment analyses, by region, are presented in S3 and S4 Tables.

#### Empowerment: By type of forage grown and small-scale irrigation practices

Our analysis sought to answer the question of whether empowerment is linked to small-scale irrigation (production and utilization, access, and control). We analyzed empowerment by disaggregating observations by decision-makers in both production and utilization of planted forages and by practices (i.e., cultivating vs. not cultivating forages, and irrigating vs. not using irrigation to produce the forages). Furthermore, with the results from Tables 3 and 4 revealing that desho and Napier grasses are the most grown and preferred forages, we assessed the linkage between empowerment and growing either Napier grass only or Napier in combination with desho grass (both scenarios potentially including other forages, as most farmers grew more than two types).

Our results (Table 9) reveal that women who had a greater say in decisions involving the production and irrigation of cultivated forages, and utilization of forages, had significantly relatively higher empowerment levels (3DE and WELI scores) than their counterparts with low levels of decision-making (i.e., women whose spouses were the dominant decision-makers) in production and irrigation of cultivated forages. Interestingly, the results show that the empowerment of the main decision-maker regarding forage utilization does appear to be linked to the sex of the decision-maker. The values for the 3DE and achieving empowerment are significantly different depending on whether the decision-maker is a man or a woman.

**Table 9:** WELI indices, for women, by forages grown and small-scale irrigation practice.

Indicator	Forages grown			Irrigation practice				
	Napier grass *(grown alone or with other forages, excluding Desho grass)*	Desho and Napier *(grown together with/without other forages)*	*t*-test	Households growing forage			Households not growing forage	
				Irrigating	Not irrigating	*t*-test^	Not irrigating	*t*-test^^
Number of observations	50	68		31	137		86	
3DE score	0.91 *(0*.*02)*	0.92 *(0*.*02)*	*t* = 0.35 df = 113 *p* = 0.03**	0.93 *(0*.*03)*	0.88 *(0*.*01)*	*t* = 16.48 df = 166 *p* = 0.00***	0.82 *(0*.*02)*	*t* = 22.80 df = 115 *p* = 0.01**
Disempowerment score (1 – 3DE)	0.09 *(0*.*02)*	0.08 *(0*.*02)*		0.07 (*0*.*03)*	0.12 *(0*.*01)*		0.18 *(0*.*02)*	
% achieving empowerment	74.00	76.92		77.42	66.42		51.16	
% not achieving empowerment	26.00	23.07		22.58	33.57		48.84	
Mean 3DE score for not yet empowered	0.65 *(0*.*02)*	0.66 *(0*.*01)*		0.67 *(0*.*02)*	0.65 *(0*.*01)*		0.63 *(0*.*01)*	
Mean disempowerment score (1 – 3DE)	0.35 *(0*.*02)*	0.34 *(0*.*01)*		0.33 *0*.*02)*	0.35 *(0*.*01)*		0.37 *(0*.*01)*	
Gender Parity Index (GPI)	0.97 *(0*.*01)*	0.98 *(0*.*01)*		0.99 *0*.*01)*	0.97 *(0*.*01)*		0.93 *(0*.*01)*	
% achieving gender parity	78.57	78.94		88.24	76.23		56.25	
% not achieving gender parity	21.43	21.05		11.76	23.77		43.75	
Average empowerment gap	0.15 *(0*.*03)*	0.11 *(0*.*02)*		0.07 *(0*.*02)*	0.15 *(0*.*02)*		0.17	
Women’s Empowerment in Livestock Index (WELI) score	0.92 *(0*.*03)*	0.93 *(0*.*02)*		0.93 *(0*.*02)*	0.89 *(0*.*01)*		0.82 *(0*.*02)*	

Note: ** level of significance at 5%; Robust standard errors of individual-level estimates are indicated in parentheses.

^Unpaired *t*-test between means of households growing forages and irrigating, and households growing forages but not irrigating.

^^ unpaired *t*-test between means of households growing forages and irrigating, and households that neither grow forages nor irrigate.

Furthermore, our analysis results (Table 9) reveal that women in households where both Napier and desho grasses were grown were significantly more empowered than women in households where no desho was grown (together with other forages). Women from households with no forages had much lower (significant) empowerment (3DE and WELI) scores than women in households growing irrigated forages. Moreover, women in households where irrigation was practiced were significantly more empowered than women in households where no irrigation was practiced. As all the households that practiced irrigation also grew forages, this result speaks to the production of small-scale irrigated forage as a composite productivity-enhancing technology and shows that women in households that practiced it were significantly more empowered than their counterparts in both households that practiced it only in part (growing forages without irrigation) and households that did not practice it (neither growing forages nor irrigating).

In terms of achieving adequacy in empowerment indicators, it is interesting to observe (Table 10) the relationship between practicing small-scale production of irrigated forages and adequacy on the “work balance” indicator. In households that practiced small-scale irrigation alongside forage production, 77% of women achieved adequacy in “work balance”; 70% of women in households that produced forages but did not practice small-scale irrigation achieved adequacy in “work balance”; only 57% of women in households that neither produced forages nor practiced irrigation achieved adequacy in “work balance”. This implies a positive difference, of about twenty percentage points (the difference between 57% and 77%), in the number of women attaining adequacy with the practice of small-scale irrigation in forage production.

**Table 10:** Percentage of women respondents achieving adequacy in WELI indicators by forages grown and small-scale irrigation practice.

Adequacy (%) by indicators	Forages grown		Irrigation practice		
	Napier grass *grown alone or with other forages, excluding Desho grass* (n = 50)	Desho and Napier *grown together with/without other forages* (*n* = 68)	Households growing forages		Households not growing forages
			Irrigating (*n* = 31)	Not irrigating (*n* = 137)	Not irrigating (*n* = 86)
Autonomy in income	10.00	55.07	35.48	41.61	36.05
Self-efficacy	62.00	78.26	83.87	63.50	65.12
Attitudes toward domestic violence	58.00	76.81	48.39	75.18	83.72
Input in productive decisions—agriculture	98.00	98.55	100.00	97.08	96.51
Input in productive decisions—livestock	90.00	98.55	87.10	94.16	95.35
Ownership of land and other assets	100.00	100.00	100.00	100.00	100.00
Access to and decisions on credit	82.00	68.12	93.55	72.26	81.40
Control over the use of income	86.00	89.86	90.32	80.29	73.26
Work balance	64.00	75.36	77.42	70.07	56.98
Visiting important locations	52.00	27.54	29.03	34.31	37.21
Group membership	100.00	100.00	100.00	100.00	98.84
Membership in influential groups	96.00	95.65	93.55	90.51	75.58
Respect among household members	90.00	84.06	90.32	78.10	54.65

Regarding the contributors to the disempowerment of women, “work balance” and “self-efficacy” did not feature as major contributors to the disempowerment of women in households where both Napier and desho grasses were grown (together with other forages). “Access to credit” did not feature as one of the major contributors to the disempowerment of women in households where no desho was grown. However, mobility constraints (“visiting important locations”) and lack of autonomy in income are the two indicators that featured as high contributors to the disempowerment of women practicing forage production and irrigation in the study sample. Moreover, although “work balance” was not the top-most constraint to empowerment, it still featured as one of the top five indicators in most categories (Table 11). The observations on indicator contribution to disempowerment are consistent with the results on the percentage of respondents achieving adequacy on the indicators (Tables 8 and 10).

**Table 11:** Indicators’ contributions to disempowerment by forages grown and irrigation practice.

Contribution to disempowerment	Forages grown		Irrigation practice		
	Napier grass *grown alone or with other forages, excluding Desho grass* (*n* = 50)	Desho and Napier *grown together with/without other forages* (*n* = 68)	Households growing forages		Households not growing forages
			Irrigating (*n* = 31)	Not irrigating (*n* = 137)	Not irrigating (*n* = 86)
Autonomy in income	0.22	0.18	0.20	0.18	0.17
Self-efficacy	0.17	0.08	0.10	0.10	0.07
Attitudes toward intimate partner violence	0.10	0.11	0.17	0.09	0.06
Input in productive decisions—agriculture	0.07	0.01	0.00	0.02	0.01
Input in productive decisions—livestock	0.01	0.01	0.07	0.03	0.01
Ownership of land and other assets	0.00	0.00	0.00	0.00	0.00
Access to and decisions on credit	0.03	0.11	0.03	0.09	0.06
Control over the use of income	0.07	0.06	0.10	0.09	0.09
Work balance	0.12	0.10	0.03	0.09	0.13
Visiting important locations	0.15	0.18	0.17	0.17	0.16
Group membership	0.00	0.00	0.00	0.00	0.00
Membership in influential groups	0.02	0.03	0.03	0.05	0.08
Respect among household members	0.03	0.11	0.10	0.10	0.15

## Discussion

Several studies have measured the empowerment of women at farm level in different ways and context. Some measured the extent and changes in empowerment [51–53] while others related empowerment with various livelihood indicators for instance household food security and nutrition [11, 39, 41, 53–57], among the later, one study [57] explored women empowerment and gender relations by looking at deviant behaviors in gender relations and shedding light on the various empowerment pathways that could be used as inputs to the design and interventions of transformative approaches. Our study is different from others on women’s empowerment as it attempts to fill the knowledge gaps in empowerment by using mixed (quantitative and qualitative) method to look at linkage between gender relations and women’s empowerment in the production and utilization of feed resources. We used quantitative and qualitative data obtained from smallholders living in the Amhara and SNNP regions, where small-scale irrigation was introduced to boost the production of cultivated forages by households [58].

Empowerment was measured within the context of livestock production using WELI. Our study findings reveal how empowerment is described by the farming community in Ethiopia. It is interesting to note that, although the word “empowerment” was used to describe the community’s aspirations for women, some of the descriptions did align with definitions of empowerment from the literature that describes empowerment as the process of gaining the (previously unattainable) capacity to exercise desired choices. When women transform resources into opportunities and economic benefits with better access to, and control over, income, their strategic choices are expanded and their economic empowerment subsequently improves [59, 60]. For example, women’s empowerment was characterized by the capacity of a woman to manage her finances and to earn money from her farm, and not always to expect a husband to provide money. However, this may come with the risk of overburdening women if the responsibility for earning income and, consequently, managing household finances is left entirely to women. Thus, women’s empowerment comes at the cost of potentially bearing the double burden of working both within and outside of the household [61], hence the need to recognize and alleviate women’s growing share of “the burden of dealing with” poverty and household responsibility [62]. That the respondents made a connection between adoption of technology (using it to improve production activities or inspiring others) with empowerment is worthy of noting. This connection is evident in literature, for instance, Banerjee et al. (2020) [54] shows that employment and income sources empower women through increased domestic decision-making power. Similarly Omondi et al. (2022) [39] points to several studies that connect knowledge (and the related increase in development outcomes) and empowerment.

Our study also assessed how women’s empowerment indices vary across various categories of households, for instance those cultivating forages and those practicing irrigation. The results reveal that small-scale irrigation technology is modestly practiced in the study area (about 18% of the households reported practicing), whereas the practice of producing planted forage was relatively common (about 66% of the study respondents indicated growing one or more types of forage crops). A majority (55%) of the farmers grew more than one forage type. Napier and desho grasses were the main fodder grown (by over 65% of the sampled farmers). The popularity of these two forage types can be attributed to their high yield potential and the fact that they can easily be propagated using cuttings and root splits (faster growth rate, ease of planting and harvesting, and high biomass, as described by the respondents). One key difference between the dominant grasses (Napier and desho) and all the other potential feeds, which was not fully explored in this paper, is that these grasses are mainly propagated by cuttings or splits, whereas most other feeds crops are propagated from seed [63]. Therefore, in addition to the cost of producing forages/feeds, part of the reason both women and men choose grasses over other alternatives could be that grasses are easily propagated from splits/runners hence no need to buy seed. Further research would be useful in understanding the implications of the costs and ease of producing different forages/feeds in the context of Ethiopia, which mostly experiences dry conditions between October to May.

It is interesting to note that comparatively more women than men indicated dependence on cultivated forages to feed their ruminant livestock, alluding to the significance of planted forages to women, who, in most cases, tend to be the ones responsible for feeding the livestock. Sales of forages were rare, and, considering the volumes produced (in terms of the percentage of land allocated to forage), this points to the implication that the volume produced was still below the threshold of producing a marketable surplus. Consequently, there still exists room for more growth in the production of planted forage and stimulating a vibrant commercialized forage value chain [63, 64]. However, the growth of fodder markets requires keen gender integration. Given the evidence, from the literature, that forage cultivation costs women additional labor and may impact on physical mobility (extra time needed to produce the fodder), a concern revealed by our study, the promotion of fodder markets without consideration of its effects on gender relations in these regions may not necessarily empower women. It may result in women losing control over the fodder once it becomes financially lucrative.

Although large cultural diversity exists within the studied regions, in the present observational study, communities in the sites revealed unique gender norms that govern access, use, and control of milk products. The most outstanding norm is the control of cow milk from own-farm production by women and that in some areas women can milk, process, market the products from milk, and control the income from milk. Norms sanction any man who attempts to take away the right from the women. Although we find that most social norms harm women, in this instance, the norm protects the rights of women. Cognizant of the interplay of other factors, development programming could benefit from such a norm considering that it supports gender inclusivity in the milk value chain. For instance, empirical evidence has shown that, when livestock production becomes commercialized, women lose control over both the livestock and the income generated, in favor of men [65–67]; however, a recent study found that income from new market opportunities (sale of surplus forage and milk) gained by households that planted forages in Ethiopia stayed under the control of women [68].

From our empowerment analysis results, the observed relatively high and comparatively significant empowerment of women involved in decision-making on irrigated forages aligned with our expectations and validated our findings. This is because the WELI, like the Women’s Empowerment in Agriculture Index (WEAI) [69], relies heavily on instrumental agency indicators, comprising mainly questions on decision-making regarding crop and livestock production, control over use of income, and decisions on financial services. It is therefore expected that decision-making over production activities would correlate highly with the empowerment index. Since the specific data used to evaluate the relationship between empowerment and decision-making on irrigated forage were not part of the data used to estimate the WELI scores (i.e., responses to the specific questions on decision-making about irrigated forages was not part of the data used to construct the index), the observation of a relatively high and comparatively significant empowerment of women involved in decision-making on irrigated forages is a clear indication that our empowerment analysis results are valid and conform to expectation.

Our results reveal that women play a critical role in forage production through decision-making and labor; for instance, the feeding of dairy cows is considered a “care role” and therefore is associated with women. Other studies have observed that women in Ethiopian rural settings play key roles in livestock husbandry and management [70]. They are most likely involved in labor-intensive roles in livestock husbandry, such as feeding, while men control the ownership, health, and marketing aspects of animal husbandry [67–69]. It is therefore salient that, from our qualitative findings of the local definitions of empowerment, we observe the linkage between empowerment and the production of forages. A common descriptor of an empowered woman encompassed hard work and strength. Moreover, having enough fodder, by cultivating and conserving feeds for livestock, was considered a vital component of empowerment for women. This can be argued to stem from the “care role”, often associated with women, which encompasses not only a duty of care for the households but also management of livestock, particularly the feeding of livestock. It is likely that a woman with enough feed, particularly during the dry season, would be considered empowered. This is from the point of view of access to resources, ability to make decisions on forage production and cultivation, and because this woman would be able to balance household chores and livestock feeding roles as opposed to spending more time gathering forage at the expense of her household chores. Consequently, forage interventions in smallholder farming systems that reduce the drudgery of labor (in production and conservation) would not only increase feed availability, but also contribute to the empowerment of women through work balance. This is an important observation, particularly for policy makers and development agents, in terms of consideration on what forage interventions to promote.

From our quantitative analysis, we observed that women from the sampled households were significantly (*p* < 0.01) less empowered (with a 3DE score of 0.87) than men (with a 3DE score of 0.91). Comparatively fewer women (63%) achieved the empowerment threshold than men (74%). Approximately 30% of women did not achieve the gender parity threshold, meaning that the men in their household were more empowered than them. This gender disparity is consistent with observations from other studies; for instance, [71] associated it with deep-rooted traditional attitudes, cultural values in the community, and a low level of literacy. That women achieved lower adequacies in “autonomy in income”, “work balance”, and “physical mobility” was not surprising considering that social norms exploration revealed that women were expected to be at home, and their physical mobility is restricted. Women were at par with men on two indicators, that is, an equal number of men and women achieved adequacy in “input in productive decisions (agriculture)” and “ownership of land and other assets”. This was expected, particularly for ownership of land as, in Ethiopia, the land policy confers equal rights on men and women, in contrast to other developing countries, where men have a clear advantage in land ownership rights. Comparatively more women than men achieved adequacy in “input in productive decisions (livestock)”, “group membership”, and “membership in influential groups”.

With the expectation that households including empowered women could have a higher propensity toward adopting new agricultural technologies and approaches, we analyzed the linkages between empowerment and practices of small-scale production of forages. We found that women were significantly more empowered in households where more types of forages were grown, where both irrigation and growing of forages were practiced, and where women had greater involvement in decision-making relating to the production and utilization of irrigated forage. These results corroborate expectations on the positive relationship between the empowerment of women and the propensity toward adopting new agricultural technologies. Our finding provides empirical evidence on the significant positive correlation between the empowerment of women and small-scale cultivation of irrigated forages. The finding answers our research question, empirically demonstrating that the empowerment of women farmers does indeed differ significantly with different gender relations dimensions, particularly access to resources and decision-making, in the context of small-scale irrigation in the production of forages.

Moreover, empirical evidence from our study, that women in households practicing small-scale production of irrigated forages had a significantly higher empowerment score and more of them were adequate in terms of “work balance” (compared with the low adequacy on the “work balance” indicator and significantly lower empowerment of women in households where less or no forage is grown and no irrigation in practiced), implies that production of more forages and the use of irrigation in the production of forages could be contributing to lessening women’s work burden. This is contrary to our expectation that the production, irrigation, and feeding of livestock roles carried out by women, in addition to other reproductive, productive, and community roles, would contribute to worsening women’s work–life balance. The determination of causal links, including the direction of causality (which would indicate whether empowerment has an impact or is a determinant in the adoption and increased use of irrigation in the small-scale cultivation of forages) is beyond the scope of our study because of a lack of available data.

However, our study results, showing the increasingly significantly different empowerment scores of women in households growing forages, growing more forage types, and practicing small-scale irrigation of the forages, in that order, point to the possibility of small-scale production of irrigated forages positively influencing the empowerment of women, and hence the possibility of shifting gender relations in farm households. This is because the order of increase in empowerment scores, from a WELI score of 0.82 for those not growing any forages, to 0.88 for those growing forages, to 0.93 for those practicing small-scale irrigation of the forages, is also the order of increase in the sophistication of the forage technology (from no use, to only growing forage, to growing forage plus irrigating it). This is another noteworthy result that could be argued to be pointing in the direction of causality, that is, that small-scale irrigation technology could explain (direction of causality) the empowerment of women in smallholder livestock keeping households, thereby contributing to the progress toward the achievement of gender equality. Alternatively, one could also hypothesize that the more empowered a household is, the more sophisticated technologies it adopts. Specifically in the context of our results, one could point to the possibility that only households with a better work–life balance can afford to adopt improved forages and irrigation (because they have time available). Although our observation corroborates the findings from other studies that the adoption of planted forages leads to net time savings for men, women, and children in cattle-rearing households [32, 68], we suggest further confirmatory studies in the form of longitudinal studies to empirically evaluate the causal pathways research and shed more light on the direction of causality between women’s empowerment and improving the availability of quality livestock feeds for smallholder farm households.

Nonetheless, we observe that “autonomy in income”, “visiting important locations” (physical mobility), and “respect among household members” were three indicators that contributed highly to the disempowerment of women farmers in households that practice small-scale irrigation to produce planted forages. This is an important result from this study that could inform strategies for achieving better equity outcomes from such feed interventions. Specifically relating to lower adequacies in the “autonomy in income” indicator as a high contributor to disempowerment of women in households growing forages and irrigating, empirical evidence that women lose control of agricultural commodities that are traditionally under their control when such commodities become financially lucrative is well known. It is intuitive that this loss of control may result in women shunning technologies that could be economically beneficial to their households. This should be an important consideration in the design of forage production interventions that are gender transformative, especially where this loss of control may also be linked to a growth in fodder markets (as pointed out earlier). Further investigation to understand the dynamics of control of income, including a hypothesis test on the link between economic benefits, division of labor, and norms, which were beyond the scope of our study, owing to data limitations, is necessary to inform the interventions.

From a global perspective, our results are consistent with patterns observed in different settings. Globally, no country has yet achieved full gender parity, with the greatest gender gaps are identified primarily in the Middle East, Africa, and South Asia [72]. Barriers to achieving gender equity generally stem from limited access to and adoption of agricultural technologies, sociocultural norms and deeply rooted beliefs about gender roles, lack of agency and lack of resources to implement policies [73]. While empirical studies consistently show that women, in agriculture, lack access to and control over resources and income, the pattern of gender disparity across countries is heterogenous [51, 74], arguably linked to socio-political values, culture, religion or family systems [74]. For instance, gender disparities in access and ownership to resources and income is likely to be more evident in farming systems of Sub Saharan Africa and South Asia, control over household income is disproportionately concentrated towards women, and women’s decision making power in agricultural production varies substantially in some South East Asian countries [74]. The inter-country heterogeneity, reinforces the need for context specific gender intervention plan to effectively eliminate gender gaps in agriculture [74].

## Conclusion

We present original research that combines quantitative and qualitative methods to reveal the relationship between different dimensions of gender relations, types of forages grown, and small-scale irrigation practice. The results of our study show that women play a dominant role in decision-making about the forages to cultivate as well as the choice of animal species to which the forages are fed. Our empirical evidence reveals the significant positive correlation between women’s empowerment and small-scale cultivation of irrigated forages. We demonstrate that the empowerment of women farmers differs significantly with different gender relations dimensions, particularly access to resources and decision-making, in the context of small-scale irrigation in the production of forages. Moreover, our study results show that production of more forages and the use of irrigation in the production of forages could contribute to lessening women’s work burden, indicating the possible contribution of small-scale irrigation technology to the progress toward the achievement of gender equality in smallholder livestock keeping households.

The results also suggest a cause–effect relationship between empowerment and small-scale production and irrigation-planted forages, and some indication of the direction of causality (the evidence from increase in number of women achieving adequacy with increased use of the technology—from just production of forages to using irrigation). As a cross-sectional study, which explored only correlations between empowerment and dimensions of gender relations, the study, by design, could not test the cause–effect relationships. We therefore recommend future causal pathways research to empirically elucidate the direction of causality between women’s empowerment and improving the availability of quality livestock feeds for smallholder farm households. This will be possible in a longitudinal study, for which the current data set could form a baseline. Moreover, a longitudinal study would clearly measure the shifts in gender relations from changes in women’s empowerment in farm households.

Nonetheless, the study reveals areas of focus that could be incorporated into the design, validation, and scaling of small-scale irrigated forage interventions not only as a way of ensuring the sustainability of the adoption of such technologies, but also as a way of contributing to the progress toward gender equality as a human development goal. As suggested by our results, strategies that would increase women’s autonomy in income and physical mobility, and build trusting relationships within the farm household, would go a long way to achieving this goal. This points to the need to integrate gender-transformative approaches in small-scale forage interventions. Empirical evidence has shown the benefit of integrating local knowledge about gender norms and other local conditions into the planning and targeting strategies for agricultural innovation [75]. We therefore argue that forage systems can achieve better outcomes by integrating gender-transformative approaches in small-scale forage interventions.

## Supporting information

S1 Table WELI indicators and definitions of adequacy cut-off. (DOCX)

S2 Table Socio-demographic characteristics and profile of decision-makers on production and irrigation of forages. (DOCX)

S3 Table WELI results by geographical location of the respondent. (DOCX)

S4 Table Percentage of respondents achieving adequacy in specific indicators and indicators’ contribution to disempowerment, by geographical location of the respondent. (DOCX)

S1 Checklist Inclusivity in global research. (DOCX)
