# Supplementary material for: Gender relations and women’s empowerment in small-scale irrigated forage production in Ethiopia
Source: PLoS One. 2024 Dec 23;19(12):e0309927. doi: 10.1371/journal.pone.0309927 (PMC11666051; doi:10.1371/journal.pone.0309927)
Supplement: S1 Table — (DOCX) [file pone.0309927.s001.docx]

**S1 Table. WELI Indicators and definitions of adequacy cut-off.**

| **Agency classification** | **Indicators (WELI subdimension)** | **Definition** |
| --- | --- | --- |
| *Intrinsic agency (Power within)* | 1. Autonomy in income | More motivated by own values than by coercion or fear of others' disapproval: Relative Autonomy Index score>=1 RAI score is calculated by summing responses to the three vignettes about a person's motivation for how they use income generated from agricultural and nonagricultural activities (yes=1; no=0), using the following weighting scheme: 0 for vignette 1 (no alternative), 2 for vignette 2 (external motivation), 1 for vignette 3 (introjected motivation), and +3 for vignette 4 (autonomous motivation) |
|  | 2. Self-efficacy | New General Self-Efficacy Scale: “Agree” or greater on average with eight self-efficacy questions |
|  | 3. Attitudes about intimate partner violence against women | Believes husband is NOT justified in hitting or beating his wife in all five scenarios: If: 1) She goes out without telling him; 2) She neglects the children; 3) She argues with him;4) She refuses to have sex with him; 5) She burns the food |
|  | 4. Respect among household members | Meets ALL the following conditions related to their spouse, the other respondent, or another household member:  1) Respondent respects relation (most of the time) &; 2) Relation respects respondent (most of the time) &; 3) Respondent trusts relation (most of the time) &; 4) Respondent is comfortable disagreeing with relation (most of the time) |
| *Instrumental agency (Power to)* | 5a. Input in productive decisions–general: | Meets at least ONE of the following conditions for ALL the agricultural activities they participate in:  1) Makes related decisions solely; 2) Makes the decision jointly and has at least some input into the decisions  3) Feels could make the decision if wanted to (to at least a MEDIUM extent) |
|  | 5b. Input in productive decisions—livestock: | Meets at least ONE of the following conditions for ALL the livestock activities they participate in:  1) Makes related decisions solely; 2) Makes the decision jointly and has at least some input into the decisions  3) Feels could make the decision if wanted to (to at least a MEDIUM extent) |
|  | 6. Ownership of land and other assets | Owns, either solely or jointly, at least ONE of the following:  1) At least THREE movable assets (equipment or consumer durables); 2) Land |
|  | 7. Access to and decisions on financial services | Meets at least ONE of the following conditions: 1) Belongs to a household that used a source of credit in the past year AND participated in at least ONE sole or joint decision about it 2) Belongs to a household that did not use credit in the past year but could have if wanted to from at least ONE source 3) Has access, solely or jointly, to a financial account |
|  | 8. Control over the use of income | Has input in decisions related to how to use BOTH income and output from ALL the agricultural activities they participate in AND has input in decisions related to income from ALL nonagricultural activities they participate in, unless no decision was made |
|  | 9. Work balance | Works less than 10.5 h per day: Workload=time spent in primary activity+(1/2) time spent in childcare as a secondary activity |
|  | 10. Ability to visit important locations | Meets at least ONE of the following conditions: 1) Visits at least TWO locations at least ONCE PER WEEK of [city, market, family/relative], or 2) Visits at least ONE location at least ONCE PER MONTH of [health facility, public meeting] |
| *Collective agency (Power with)* | 11. Group membership | An active member of at least ONE group |
|  | 12. Membership in influential groups | An active member of at least ONE group that can influence the community to at least a MEDIUM extent |
