# Supplementary material for: Gender relations and women’s empowerment in small-scale irrigated forage production in Ethiopia
Source: PLoS One. 2024 Dec 23;19(12):e0309927. doi: 10.1371/journal.pone.0309927 (PMC11666051; doi:10.1371/journal.pone.0309927)
Supplement: S2 Table — (DOCX) [file pone.0309927.s002.docx]

**S2 Table: Socio-demographic characteristics and profile of decision-makers on production and irrigation of forages.**

| Characteristics | | **Decision-maker 1** | | | **Decision-maker two** | | | **Decision-maker three** | | |
| --- | --- | --- | --- | --- | --- | --- | --- | --- | --- | --- |
|  |  | **Amhara** | **SNNP** | **Overall** | **Amhara** | **SNNP** | **Overall** | **Amhara** | **SNNP** | **Overall** |
| Age | | 37.6 | 40.9 | 39.8 | 44.6 | 48.4 | 47 | 17 | 25.2 | 22.4 |
| Schooling | University | 1. | 1.1 | 1.1 | 3.1 | 13.1 | 9.6 | 1.2 | 6 | 4.4 |
|  | Above grade 8 | 15.3 | 19.9 | 18.23 | 11.5 | 34.1 | 26.1 | 47.6 | 53.3 | 51.4 |
|  | Grade 1–8 | 21.4 | 52.85 | 41.6 | 39.6 | 45.5 | 43.4 | 40.3 | 29.4 | 32.9 |
|  | No formal schooling | 62.2 | 23.9 | 37.6 | 38.5 | 6.3 | 17.7 | 11 | 10.8 | 10.8 |
|  | Adult literacy | 0 | 2.3 | 1.5 | 7.3 | 1.1 | 3.3 | 0 | 0.6 | 0.4 |
| Full adult workload | Yes | 97.5 | 96.4 | 96.8 | 92.7 | 86.3 | 88.4 | 57.5 | 65.3 | 62.7 |
|  | No | 2.5 | 3.6 | 3.2 | 7.3 | 13.7 | 11.6 | 47.5 | 37.4 | 6.7 |
| Residence | Mostly at home | 100 | 93.5 | 96 | 87.8 | 67.9 | 74.4 | 56.3 | 64.1 | 61.7 |
|  | Traveling | 0 | 6.6 | 4.4 | 11 | 22 | 18.4 | 0 | 20.6 | 13.6 |
|  | Formal employment | 0 | 0 | 1.9 | 1.2 | 7.1 | 5.2 | 0 | 4.1 | 2.7 |
|  | others | 0 | 0 | 0 | 0 | 3 | 2 | 43.7 | 11.2 | 22 |
