# Supplementary material for: Gender relations and women’s empowerment in small-scale irrigated forage production in Ethiopia
Source: PLoS One. 2024 Dec 23;19(12):e0309927. doi: 10.1371/journal.pone.0309927 (PMC11666051; doi:10.1371/journal.pone.0309927)
Supplement: S3 Table — (DOCX) [file pone.0309927.s003.docx]

**S3 Table*:* WELI results by geographical location of the respondent.**

| **Indicators** | **Study geographical regions** | | |
| --- | --- | --- | --- |
|  | **Amhara** | **SNNP** | **T-test** |
| Number of observations | 82 | 168 |  |
| 3DE score | **0.87** ***(0.02)*** | **0.86**  ***(0.01)*** | t=0.50 df=248  *p=0.02*** |
| Disempowerment score (1–3DE) | 0.13 *(0.02)* | 0.14  *(0.01)* |  |
| % achieving empowerment | 66.00 | 62.00 |  |
| % not achieving empowerment | 34.00 | 38.00 |  |
| Mean 3DE score for not yet empowered | 0.63  *(0.02)* | 0.64  *(0.01)* |  |
| Mean disempowerment score (1–3DE) | 0.37  *(0.02)* | 0.36  *(0.01)* |  |
| Gender Parity Index (GPI) | 0.95  *(0.01)* | 0.96  *(0.01)* |  |
| % achieving gender parity | 0.67 | 0.71 |  |
| % not achieving gender parity | 0.33 | 0.29 |  |
| Average empowerment gap | 0.16  *(0.03)* | 0.15  *(0.01)* |  |
| WELI score | 0.88  *(0.02)* | 0.87  *(0.01)* |  |

Note: Robust standard errors of individual-level estimates are indicated in parenthesis
