# Supplementary material for: Gender relations and women’s empowerment in small-scale irrigated forage production in Ethiopia
Source: PLoS One. 2024 Dec 23;19(12):e0309927. doi: 10.1371/journal.pone.0309927 (PMC11666051; doi:10.1371/journal.pone.0309927)
Supplement: S4 Table — (DOCX) [file pone.0309927.s004.docx]

**S4 Table: Percentage of respondents achieving adequacy in specific indicators and indicators’ contribution to disempowerment, by geographical location of the respondent**

|  | **Percentage of respondents achieving adequacy** | | **Contributions to disempowerment (proportions)** | |
| --- | --- | --- | --- | --- |
| **Adequacy (%) by indicators** | **Amhara**  **(n=82)** | **SNNP**  **(n=168)** | **Amhara (n=82)** | **SNNP**  **(n=168)** |
| Autonomy in income | 17.07 | 50.60 | 0.19 | 0.17 |
| Self-efficacy | 48.78 | 75.60 | 0.15 | 0.06 |
| Attitudes toward domestic violence | 65.85 | 79.76 | 0.10 | 0.06 |
| Input in productive decisions—agriculture | 93.90 | 98.81 | 0.03 | 0.01 |
| Input in productive decisions—livestock | 89.02 | 97.02 | 0.05 | 0.01 |
| Ownership of land and other assets | 100.00 | 100.00 | 0.00 | 0.00 |
| Access to and decisions on credit | 92.68 | 69.05 | 0.03 | 0.09 |
| Control over the use of income | 84.15 | 75.60 | 0.07 | 0.10 |
| Work balance | 50.00 | 74.40 | 0.15 | 0.09 |
| Visiting important locations | 62.20 | 22.02 | 0.13 | 0.18 |
| Group membership | 100.00 | 99.40 | 0.00 | 0.00 |
| Membership in influential groups | 92.68 | 82.14 | 0.03 | 0.08 |
| Respect among household members | 84.15 | 66.07 | 0.07 | 0.14 |
